# Investigation on size tolerance of pore defect of girth weld pipe

**DOI:** 10.1371/journal.pone.0191575

**Published:** 2018-01-24

**Authors:** Yan Li, Jian Shuai, Kui Xu

**Affiliations:** Department of Mechanical and Transportation Engineering, China University of Petroleum-Beijing, Beijing, China; University of Michigan, UNITED STATES

## Abstract

Welding quality control is an important parameter for safe operation of oil and gas pipes, especially for high-strength steel pipes. Size control of welding defect is a bottleneck problem for current pipe construction. As a key part of construction procedure for butt-welding of pipes, pore defects in girth weld is difficult to ignore. A three-dimensional non-linear finite element numerical model is established to study applicability of size control indices based on groove shape and softening phenomenon of material in heat-affected zone of practical pipe girth weld. Taking design criteria of pipe as the basis, basic tensile, extremely tensile and extremely compressive loading conditions are determined for pipe stress analysis, and failure criteria based on flow stress is employed to perform stress analysis for pipe girth weld with pore defect. Results show that pipe girth welding stresses of pores at various radial locations are similar. Whereas, stress for pores of different sharpness varied significantly. Besides, tolerance capability of API 5L X90 grade pipe to pore defect of girth weld is lower than that of API 5L X80 grade pipe, and size control index of 3 mm related to pore defect in current standards is applicable to API 5L X80 and X90 grade girth welded pipes with radially non-sharp pore defects.

## Introduction

Girth welding is a key procedure used in pipe construction, primarily influenced by welding technology and external environments [1]. During melting and solidification of metals, some gases are inevitably retained in the metal, resulting in the formation of pore defects [2–4]. Pore defects are a typical volumetric defect observed in pipe girth welds. These defects reduce actual cross-sectional area, quality of air seal [3–4], decrease material strength and toughness [5–7], increase local stress concentration, and reduce stiffness of girth weld. This is especially the case when size of pore defects exceeds a certain threshold. As a result, failure may be induced along the cross-section of pore and safe operation of pipe is threatened [7–9]. Therefore, it is vital to determine quality control indices for the weld based on effect of pore defect on the stress profile of the pipe girth weld structure.

Forming mechanism and control measures are major focus of current investigations of weld pore defects [10–13]. In small-scale tests, welding can be interrupted by an artificially implanted defect, allowing formation of a pore defect at specific conditions [14]. However, stress distribution around a pore defect cannot be empirically observed. To accurately describe stress distribution around pore defects, finite element simulation was carried out to study effect of microscopic structural defects on macroscopic mechanical behaviors [15–18]. In research reported in literature, investigations are limited in discussions about slag and pore defects at microscopic scale. Domestic and foreign standards API Std 1104 and SY/T 4109 for pore defects in pipe welds primarily prescribe size control. However, in the case of long distance pipe development with a large diameter, thin wall and high pressure, use of high-grade pipe steel requires a higher standard of quality control for the pipe girth weld [19]. Xiong et al. analyzed effects of defect size and matching factor of weld strength by two-dimensional finite element model for weld defects in high strength pipes [20]. However, two-dimensional model was found to be insufficient and did not accurately reflect three-dimensional characteristics of pore defects in the pipe with a girth weld. Therefore, stress analysis is a critical need for high-strength girth welded pipe with pore defects.

Quality control for high strength girth welded pipe in enabling pipe safety is important. Additionally, research reported in literature rarely takes stress analysis of girth welded pipes with pore defects in account. As a result, it can be concluded that technological foundation for pore defect size control of girth weld in high strength pipe is currently lacking. The present work concentrates on groove shape and softening phenomenon of material in heat-affected zone of the pipe girth weld in practical engineering applications. Moreover, a numerical model for pipe girth weld with pore defect is established. Effects of pore defects based on stress distribution of pipe girth weld from simulation results for three loading conditions are analyzed. Effects of location and shape factor of pores on high-grade pipe are studied. Additionally, applicability of pore defect size control indices in current standards are evaluated. Based on results, some guidelines for reasonable control of welding quality of pipe girth weld s are discussed.

## Finite element model

### Welding groove size and shape

**Fig 1:**
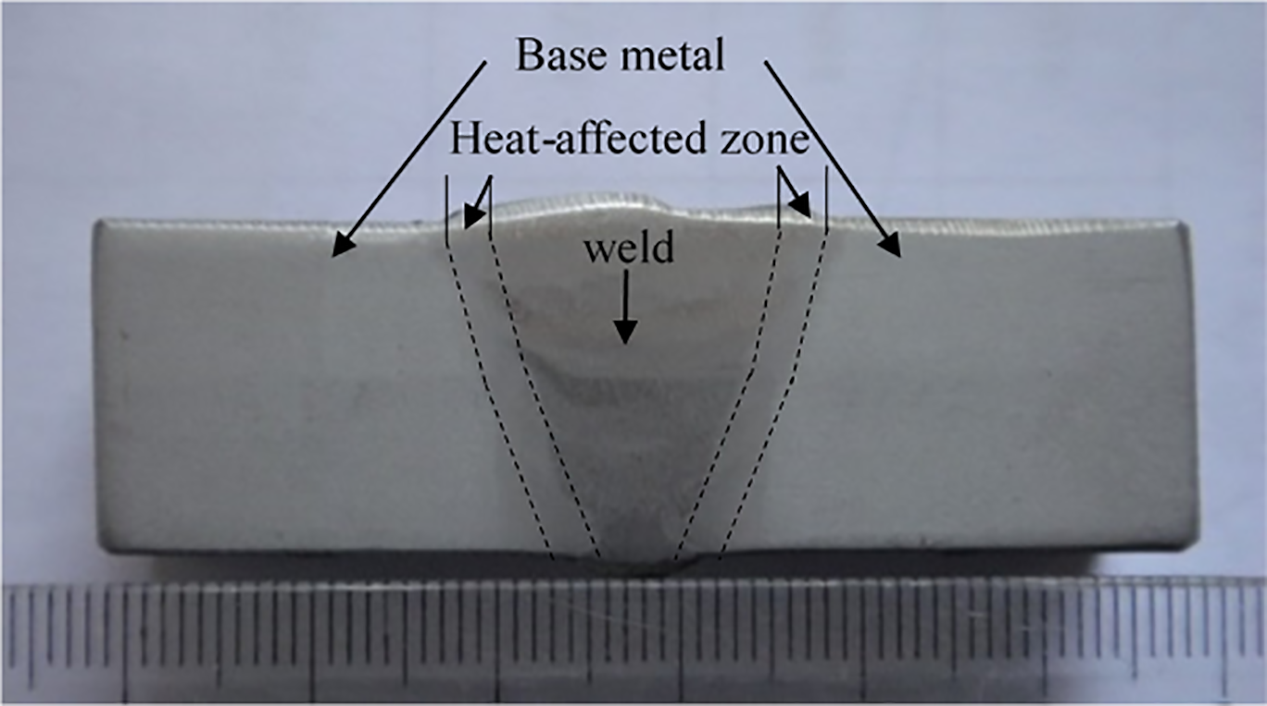
Cross section of weld.

**Fig 2:**
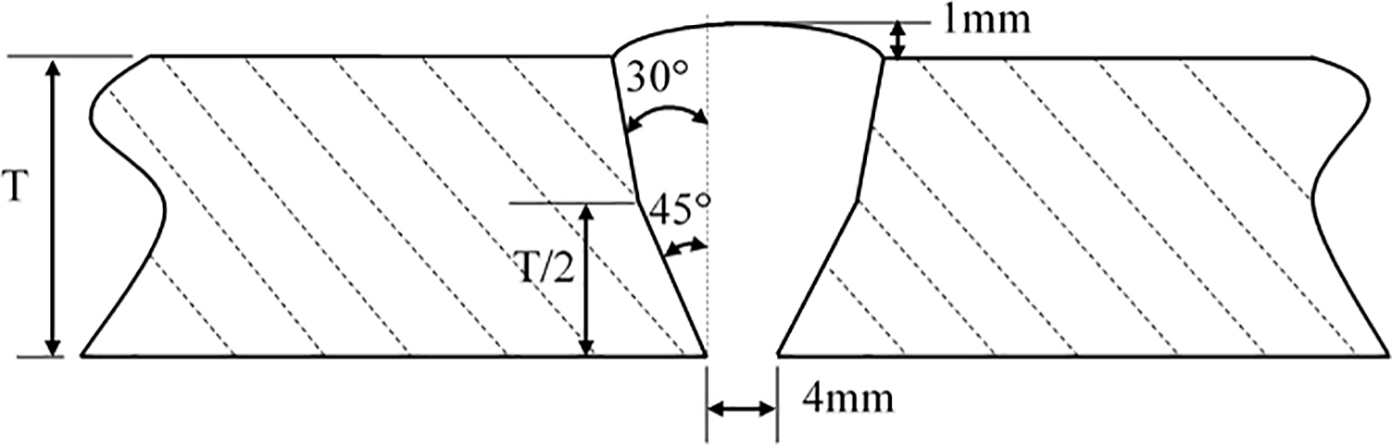
Size of weld.

Pipes simulated in the present work are modeled as in-service pipeline. The welding process is Flux-Cored Arc Welding (FACW) which has been successfully applied in API 5L X80 grade gas pipeline in China. The welding process combined with low-hydrogen electrode for root welding, self-shielded flux cored arc welding for semi-automatic filling and capping. The root welding material is AWS A5.1 E7016 low-hydrogen electrode, the filling and capping material is AWS A5.29 E81T8 self-shielded flux cored arc welding. Pipe dimensions are Φ 1219×16.3 mm for API 5L X90 grade pipe and Φ 1016×18.4 for API 5L X80 grade pipe, respectively. Fig 1 shows the real cross section shape of weld specimen cut from the in-service pipeline and its corresponding sizes are shown in Fig 2. It can be observed from the sectional view that the material is divided into multiple zones. This is primarily because the material receives a high heat input during the welding process. As a result, material grain structure and performance are changed remarkably and division of material occurs [21]. In the Fig 2, the middle part is the weld zone formed by melting and solidification between filler metal and base metal. Two side parts are base metal unaffected by the welding process. Finally, a heat-affected zone is formed by the base metal between the girth weld and base metal that is close to the weld and is heated by the high temperature of weld metal. According to the groove size shown in Fig 1, width of the weld root at the border of the base metal on the bottom of weld is 4 mm. Width of the weld toe at the border of the base metal on the top of weld is 16 mm. Average width of the weld root and toe for the heat affected zone is approximately 3 mm. On the weld surface, height of deposited metal above the connection line of the weld toe is 1 mm. This is the remaining height that increases cross-sectional area of weld and enhances load carrying capacity, and it is taken into account in analysis in the present work. US standard API Std 1104 prescribes size of a single pore defect to be a maximum size of 3 mm and 25% of pipe wall thickness. Whereas, Chinese standard SY/T 4109 draws on API standard requirements by limiting diameter of a single pore at a maximum of 3 mm. Based on both the US and Chinese standards, pore diameter in the present work is specified as 3 mm.

### Mesh generation

**Fig 3:**
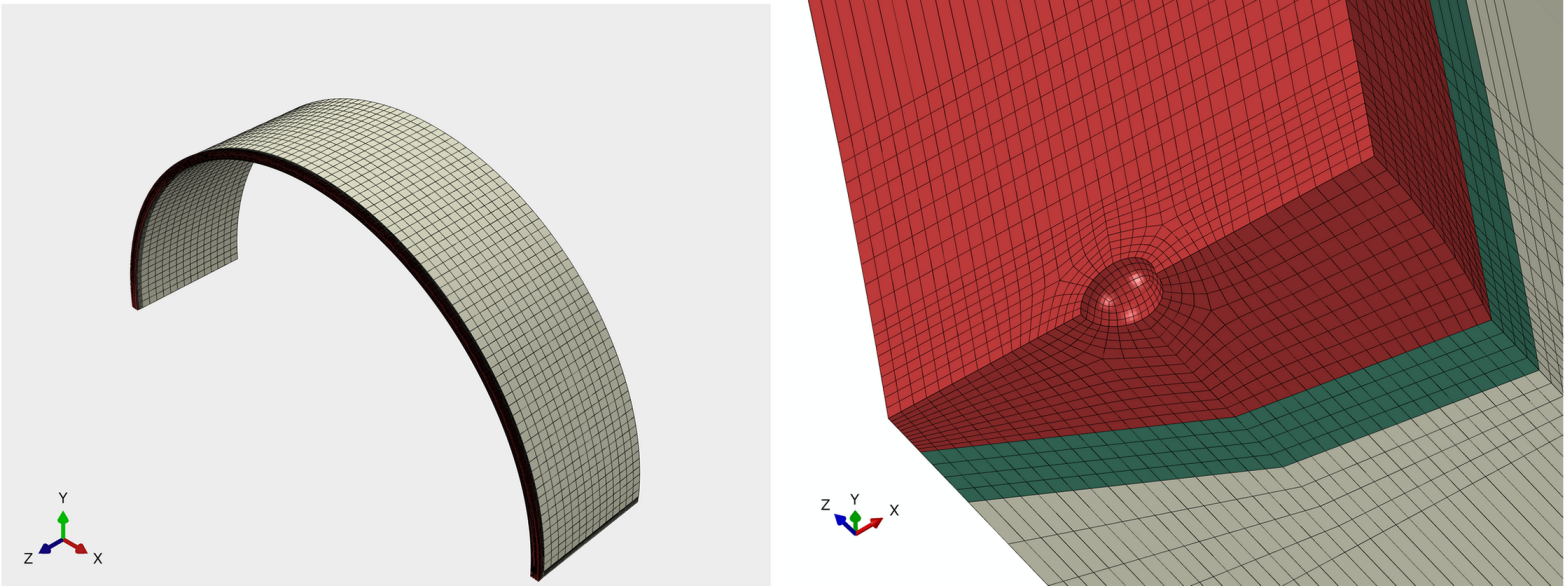
Finite element model. (A) The whole pipe. (B) Local area of pore defect.

Here, a large-scale finite-element analysis (FEA) was carried out using commercial FEA software ABAQUS. Pore defect is approximated as a regular sphere located at the center of the pipe girth weld. Since, the pipe is symmetrical about the horizontal and vertical cross sections where the pore center is located, 1/4 of the pipe is used to carry out the modeling and simulation. Hexahedral elements with 20 nodes are used for model meshing. Fine meshing is adopted around the pore as stress gradients vary around the pore. Coarse mesh is used for the pipe, as the distance from the pore is increased. The finite element model is shown in Fig 3.

### Material parameter

During welding, the material is subjected to multiple thermal cycles, causing change in mechanical behavior of the materials in the weld or adjacent to the base material. In practice, matching factor of weld strength is used to characterize uneven change of material caused by welding, and this factor is typically obtained empirically. To understand strength matching for API 5L X80 and X90 grade pipes with girth weld, several pipe girth welded samples are tested and measured. This allows changes to material property of the weld, base material and heat-affected zone to be characterized. Test results show that yield limit of the weld is 10% to 12% higher than that of the base material, demonstrating overmatched welding. Welding with this factor can increase the stiffness of girth weld. However, microscopic structure is changed and a softened zone is formed in the heat-affected zone due to welding thermal cycles, causing decreased strength and toughness [22]. Compared to the base material, yield limit of this part is reduced by 2% to10%.

Based on matching relationship of weld strength obtained in the tensile tests on the static universal testing machine, calculations were carried out with overmatching factor of 1.1, i.e. strength of the weld zone material is 10% higher than the base material, and that of the heat-affected zone material is 10% lower. Material stress-strain relationship is described by Ramberg-Osgood (R-O) constitutive relation, as shown in Eq (1).

$$\frac{\varepsilon }{\varepsilon_{s}}=\frac{\sigma }{\sigma_{s}}+\alpha {\left(\frac{\sigma }{\sigma_{s}}\right)}^{n}$$ (1)

where, *ε* is true strain, *σ* is true stress in MPa, *E* is Young’s modulus in MPa, *σ*_*s*_ is yield limit in MPa, *α* is offset of yield, *n* is hardening exponent, and *ε*_*s*_ is elastic strain corresponding to yielding, *ε*_*s*_ = *σ*_*s*_/*E*.

Grade of pipe is API 5L X80 or X90. Yield limit *σ*_*s*_ and strength limit *σ*_*b*_ in MPa are specified as lowest values prescribed by API-5L. In uniaxial tensile test, when the material reaches its strength limit, tensile load is given as:

dF=d(σ⋅A)=σdA+Adσ=0 (2)

where, *F* is tensile load in tensile test N, *A* is cross-sectional area of test sample in mm, and *σ* is true stress of test sample in MPa.

Eq (2) can be converted to

$$\frac{d\sigma }{\sigma }=\frac{dA}{A}$$ (3)

Due to volume conservation law, the following equation is obtained:

$$\frac{d\sigma }{\varepsilon }=\sigma$$ (4)

By substituting the equation above into Eq (1), parameters *α* and *n* can be determined based on yield limit *σ*_*s*_ and ultimate limit *σ*_*b*_. Material properties of all the parts are shown in Table 1.

**Table 1:** Parameters of the Ramberg-Osgood model.

Pipe grade	Material type	*σ*_*s*_/MPa	*σ*_*b*_/MPa	*E*/MPa	*α*	*n*
**API 5L X80**	Heat affected zone	499.5	562.5	200000	1.00	23.74
	Base material	555	625	200000	0.80	24.47
	Weld	610.5	687.5	200000	0.64	25.31
**API 5L X90**	Heat affected zone	562.5	625.5	200000	0.78	26.72
	Base material	625	695	200000	0.6	27.79
	Weld	687.5	764.5	200000	0.45	29.08

## Loading and boundary conditions of girth weld

Loading conditions need to be established to perform stress analysis for pipe girth weld with pore defects. First, for calculations, internal pressure of the designed pressure level is decided as the basic loading. This is because design pressure is the maximum possible pressure during operation. Second, according to design code for pipes GB50251, maximum shear stress theory of failure is used to perform strength check, and strength condition is:

$$\sigma_{T}=\sigma_{1}-\sigma_{3}⩽0.9\sigma_{s}$$ (5)

Where, *σ*_*T*_ denotes equivalent stress of Tresca strength theory in MPa, *σ*_1_ denotes maximum principal stress in MPa and *σ*_3_ denotes minimum principal stress in MPa.

**Fig 4:**
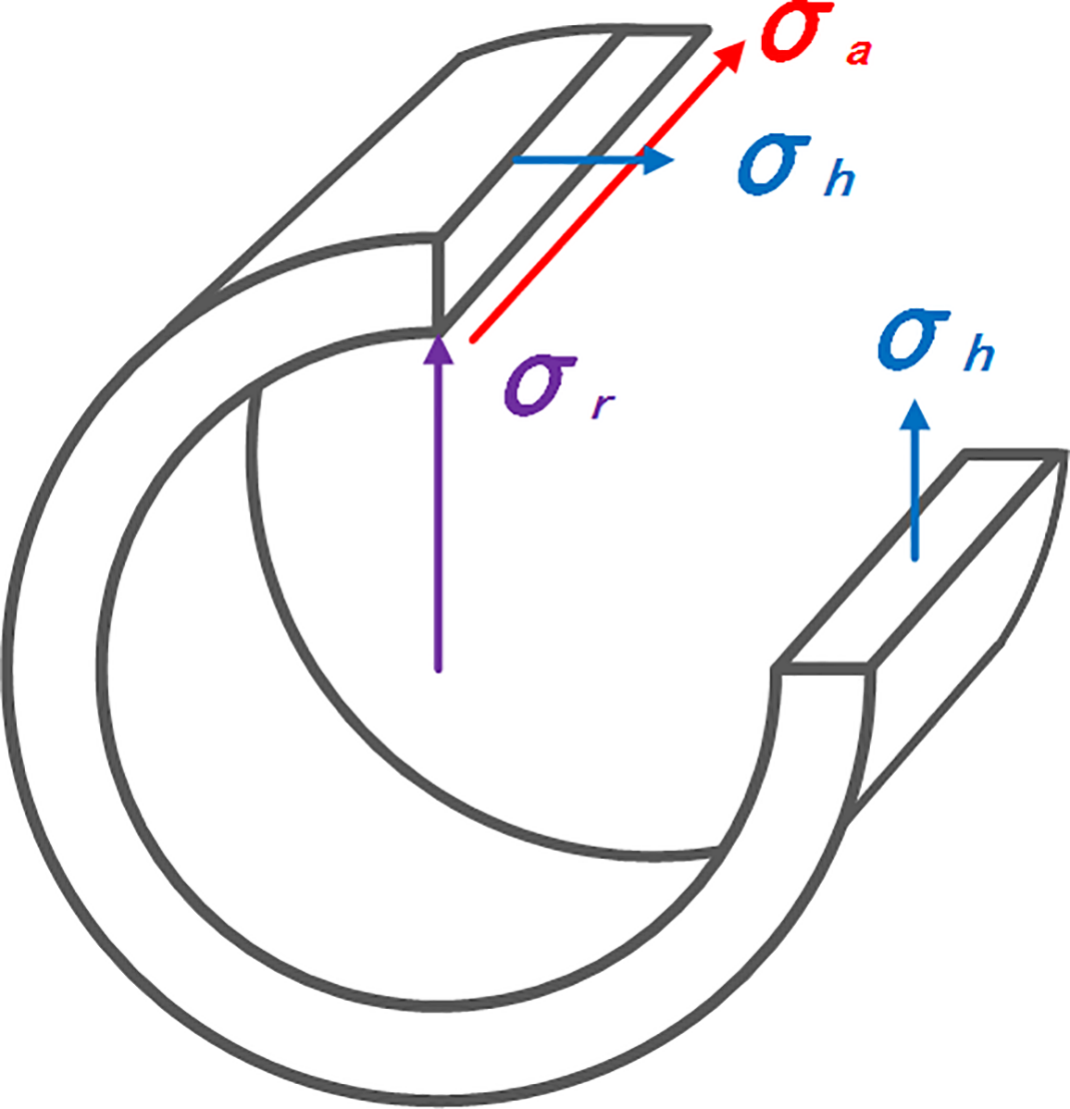
Stress state of pipe.

In fact, the thickness of pipe is much less than diameter, so the radial stress (*σ*_*r*_) can be reasonably ignored. Consequently, the pipe is subjected to biaxial stress, hoop stress (*σ*_*h*_) and axial stress (*σ*_*a*_), as shown in Fig 4. The internal pressure induces circularly the tensile stress in MPa, and axial stress in MPa, which is either compressive or tensile. Under design internal pressure, the hoop stress reaches 0.72 *σ*_*s*_. Thus, extreme tensile and compressive loading conditions situations under basic loading can be determined when axial stress of pipe is compressive (*σ*_*a*_<0), by:

$$\sigma_{1}=\sigma_{h}=0.72\sigma_{s},\;\sigma_{3}=\sigma_{a}$$ (6)

$$\sigma_{T}=\sigma_{1}-\sigma_{3}=\sigma_{h}-\sigma_{a}⩽0.9\sigma_{s}$$ (7)

Corresponding axial stress is:

$$\sigma_{a}⩾\sigma_{h}-0.9\sigma_{s}=‑0.18\sigma_{s}$$ (8)

i.e. extreme loading condition of pipe axial compression is superposition of the design pressure and an axial compressive stress 0.18 *σ*_*s*_.

When, axial stress of pipe is tensile (*σ*_*s*_ >0),

$$\sigma_{1}=\sigma_{a},\;\sigma_{2}=\sigma_{h}=\sigma_{a}⩽0.9\sigma_{s},\;\sigma_{3}=0$$ (9)

$$\sigma_{T}=\sigma_{1}-\sigma_{3}=\sigma_{a}⩽0.9\sigma_{s}$$ (10)

i.e. the extreme loading condition of pipe axial tension is combination of the design pressure and an axial tensile stress 0.9 *σ*_*s*_.

Based on the discussions above, stress analysis is performed under the following three loading conditions:

1. Basic loading condition: design pressure of 12 MPa;
2. Extreme compressive loading conditions: superposition of design pressure of 12 MPa and axially compressive stress 0.18 *σ*_*s*_;
3. Extreme tensile loading conditions: superposition of design pressure of 12 MPa and axially tensile stress 0.9 *σ*_*s*_.

This research considers two conditions, pipe subjected to internal pressure only and pipe subjected to internal pressure combined with axial pressure. Loads in each model were applied as monotonically increasing way, where internal pressure remains normal to the pipe internal surface and axial pressure remains normal to the pipe end. Boundary conditions were applied in cylindrical coordinate system. Two symmetric displacement constraints were applied to the quarter models, in the cross and axial section planes. In order to eliminate the effect of rigid displacement of models, hoop displacement constraint was applied on pipe end, which can be seen in Fig 3.

## Failure criteria

Reasonable determination of failure criteria requires consideration of stress state and material properties. From the perspective of stress state, pore defects are initial defects generated in production, and existence of such a defect inevitably results in increased local stress in the weld zone. For a pipe subjected to multi-axial stress states, failure cannot be determined by a single stress component, but is influenced by multi-axial stress. As a result, strength theory for multi-axial stress states should be applied. In conventional strength theories, von Mises strength theory considers influence of 3 principal stresses, which is in line with test results. Thereby, present work performs judgment by using von Mises equivalent stress.

From the perspective of material properties, during plastic deformation, stress-strain relationship of material is characterized by nonlinear change. As plastic deformation increases, capability to resist plastic deformation is also elevated. Hahn et al. considered this strain hardening effect and proposed the concept of flow stress [23]. Due to this, upper and lower bounds of flow stress are defined as strength limit and yield limit of material, respectively based on stress levels. Especially for modern high-grade pipes, improvement of material properties enhances both strength and strain hardening capabilities of the pipe. Besides, weld plays a role of reinforcement to some extent in pipes with overmatched weld. Therefore, according to the definitions of upper and lower bounds of flow stress, flow stress is specified as average of yield strength and strength limit of weld material. Flow stress can be effectively utilized to evaluate pipe girth weld with pore defects using failure criteria. This is based on reinforcement effect of overmatched weld material to ensure sufficient safety of pipe girth weld.

## Result

### Stress analysis

**Fig 5:**
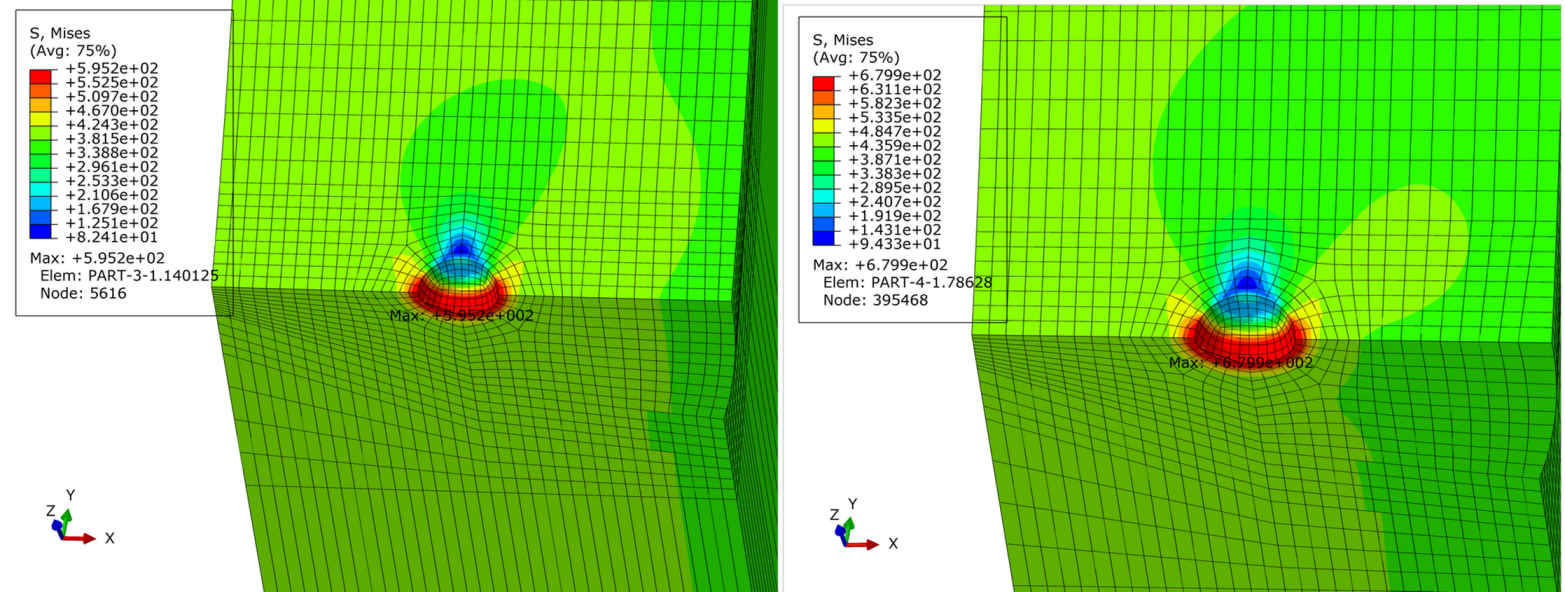
von Mises stress distribution of pipe under basic loading condition. (A) API 5L X80 grade pipe. (B) API 5L X90 grade pipe.

**Fig 6:**
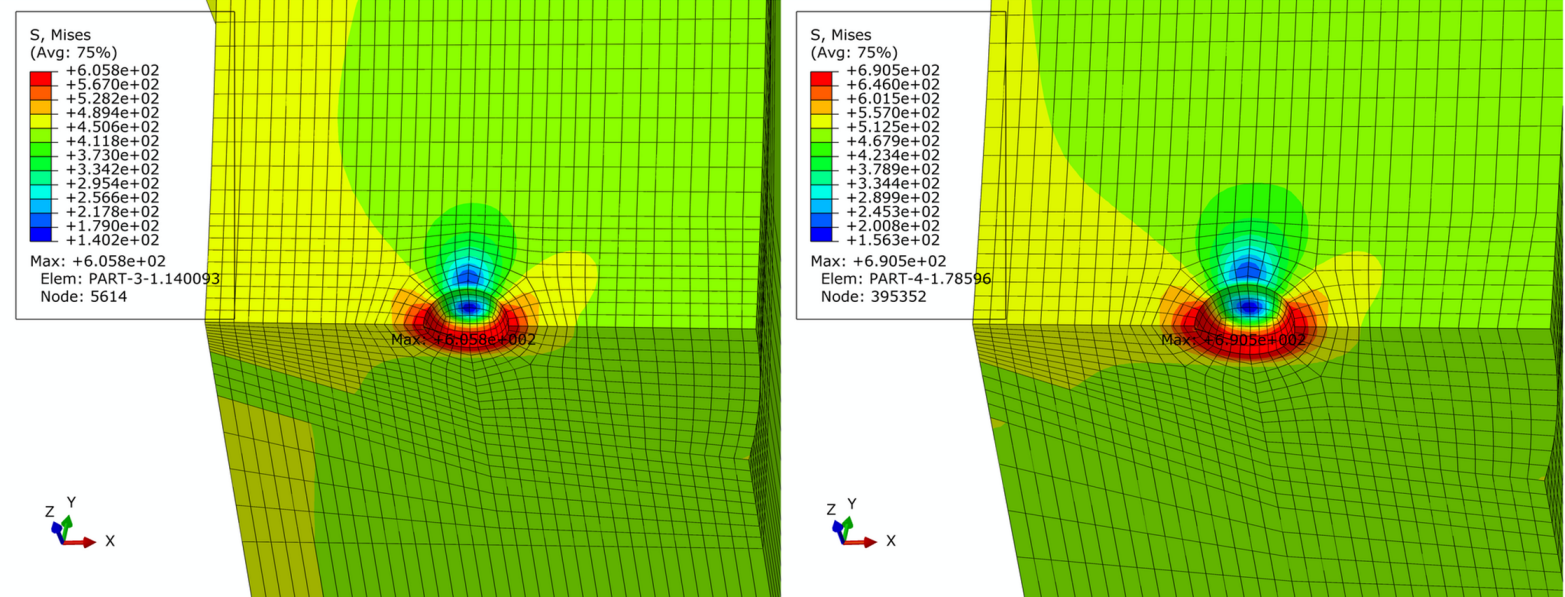
von Mises stress distribution of pipe under extremely compressive loading condition. (A) API 5L X80 grade pipe. (B) API 5L X90 grade pipe.

**Fig 7:**
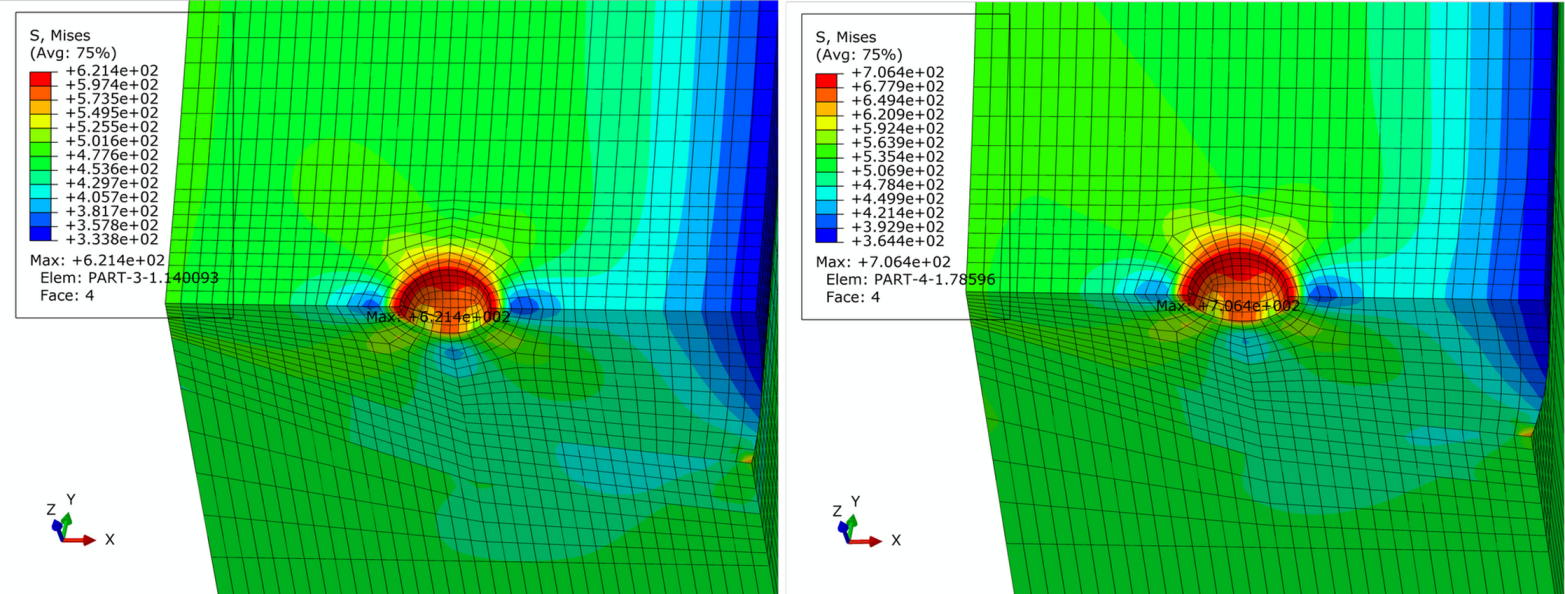
von Mises stress distribution of pipe under extremely tensile loading condition. (A) API 5L X80 grade pipe. (B) API 5L X90 grade pipe.

For basic, extremely compressive and tensile loading conditions determined in Section 2, finite element simulation of pipe is performed. Figs 5–7 show contours of von Mises equivalent stress distribution around the pore defect under the three loading conditions, respectively. It can be observed from the figures that the pore induces significantly increased stress near the pore, but influence is limited in close vicinity around the pore without extension in to inner or outer sides of the pipe girth weld. Additionally, stress field is evenly distributed in the remaining area of pipe; under none of the three loading conditions is yielding reached. When the pipe is subjected to a design pressure of 12 MPa under basic loading, internal pressure causes expansion of pipe. Maximum equivalent stress under these conditions is located near the pore on the vertical plane of symmetry of pipe. When the pipe is subjected to extremely axially compressive loading that is the combination of design pressure of 12 MPa and axial compressive stress equal to 0.18 times that of yield strength, due to expansion of pipe caused by combination of axially compressive loading and internal pressure, maximum equivalent stress increases by a small margin with location remaining unchanged near the pore on the vertical plane of symmetry. When the pipe is subjected to the extremely axially tensile loading that is the combination of design pressure of 12 MPa and axial tensile stress equal to 0.9 times that of yield strength, due to overwhelming axial tensile stress on the pipe with respect to hoop stress caused by internal pressure, location of maximum equivalent stress in pipe moves to the edge of pore on the cross section. Maximum equivalent stress of pipe and average stress of the far end of pipe body under each loading condition is listed in Table 2. Based on the failure criteria determined above, none of the maximum von Mises equivalent stresses for the three loading conditions exceeded the corresponding flow stress described in the failure criteria. Meanwhile, except for the area where local stress around the pore is increased, stress level of the remaining pipe body is within the elastic range. This demonstrates that the sphere pore defect with a diameter of 3 mm is acceptable for API 5L X80 and X90 grade pipes with a girth weld.

**Table 2:** The von Mises stress of pipe.

Loading condition	Pipe grade	von Mises stress/MPa		Flow stress/MPa
		Pipe with pore	Intact pipe	
**Basic**	API 5L X80	595.2	398.8	649
	API 5L X90	679.9	450.2	726
**Extremely compressive**	API 5L X80	605.8	454.0	649
	API 5L X90	690.6	513.2	726
**Extremely tensile**	API 5L X80	621.4	452.8	649
	API 5L X90	706.4	510.6	726

### Location of pore

**Fig 8:**
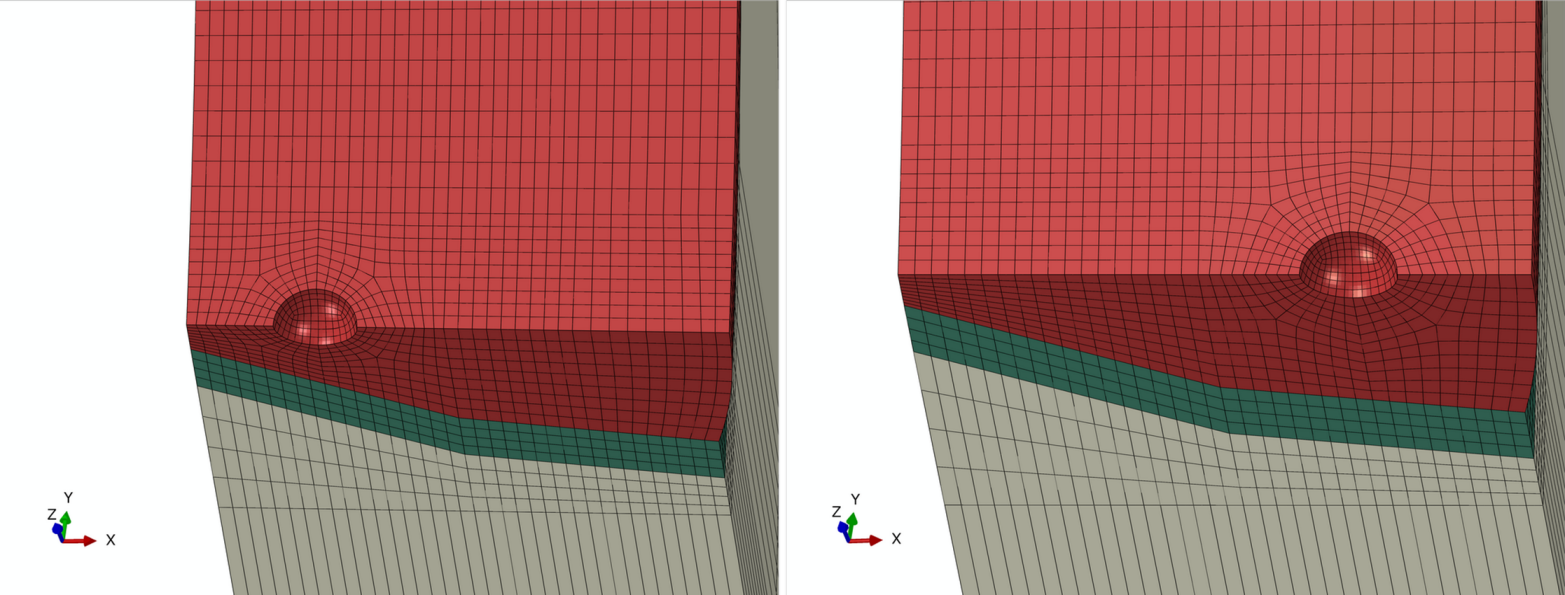
Finite element model of pore with different location. (A) inner side. (B) outer side.

**Fig 9:**
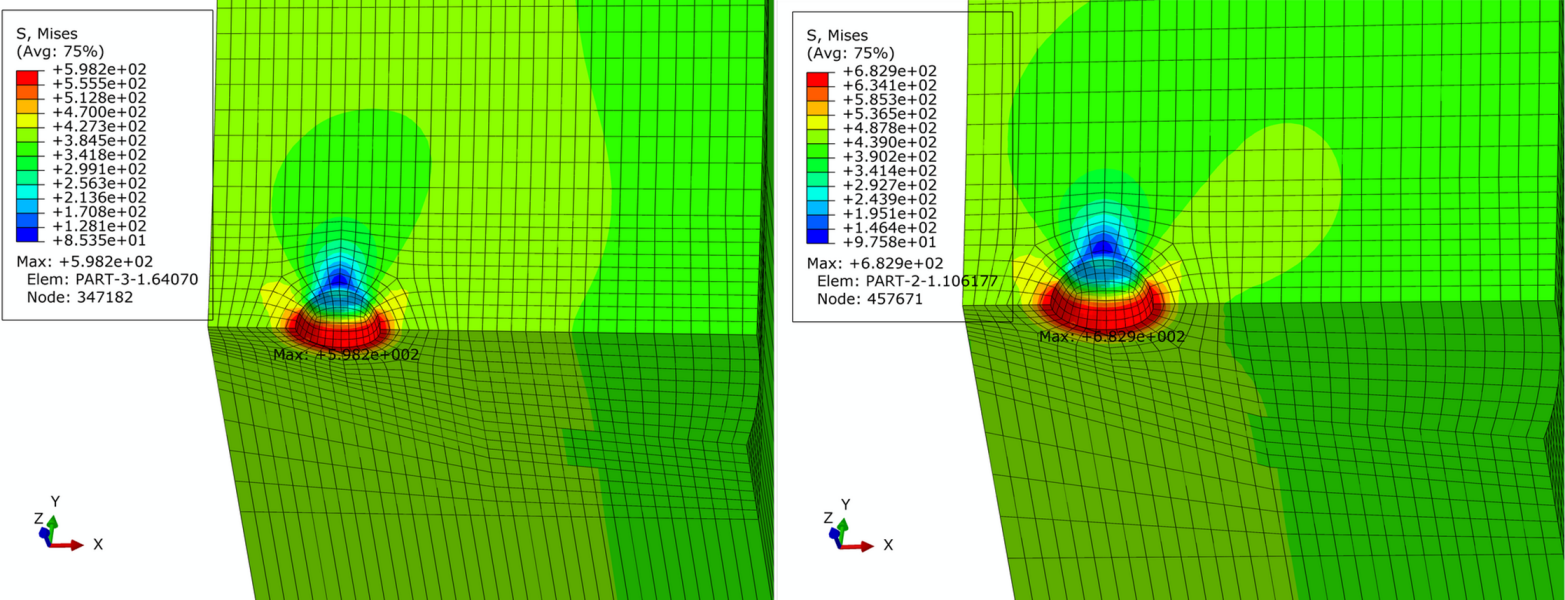
von Mises stress distribution of pipe with inner pore under basic loading condition. (A) API 5L X80 grade pipe. (B) API 5L X90 grade pipe.

**Fig 10:**
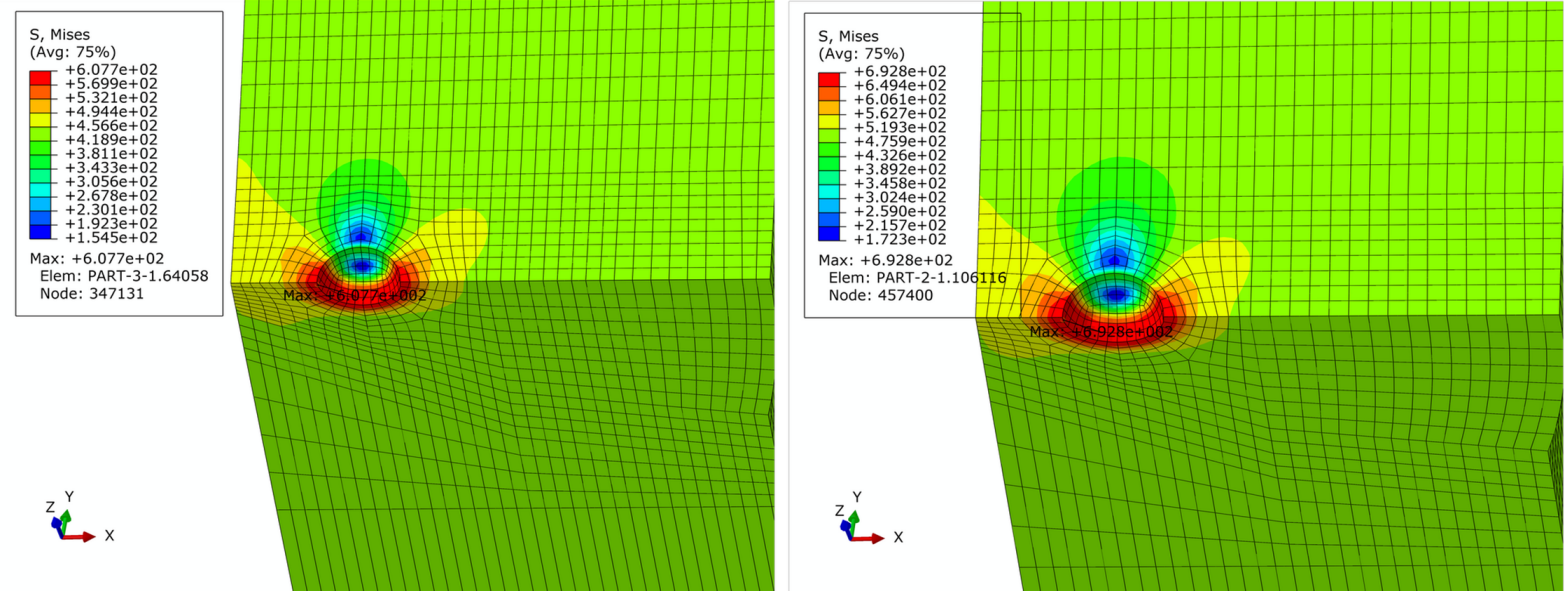
von Mises stress distribution of pipe with inner pore under extremely compressive loading condition. (A) API 5L X80 grade pipe. (B) API 5L X90 grade pipe.

**Fig 11:**
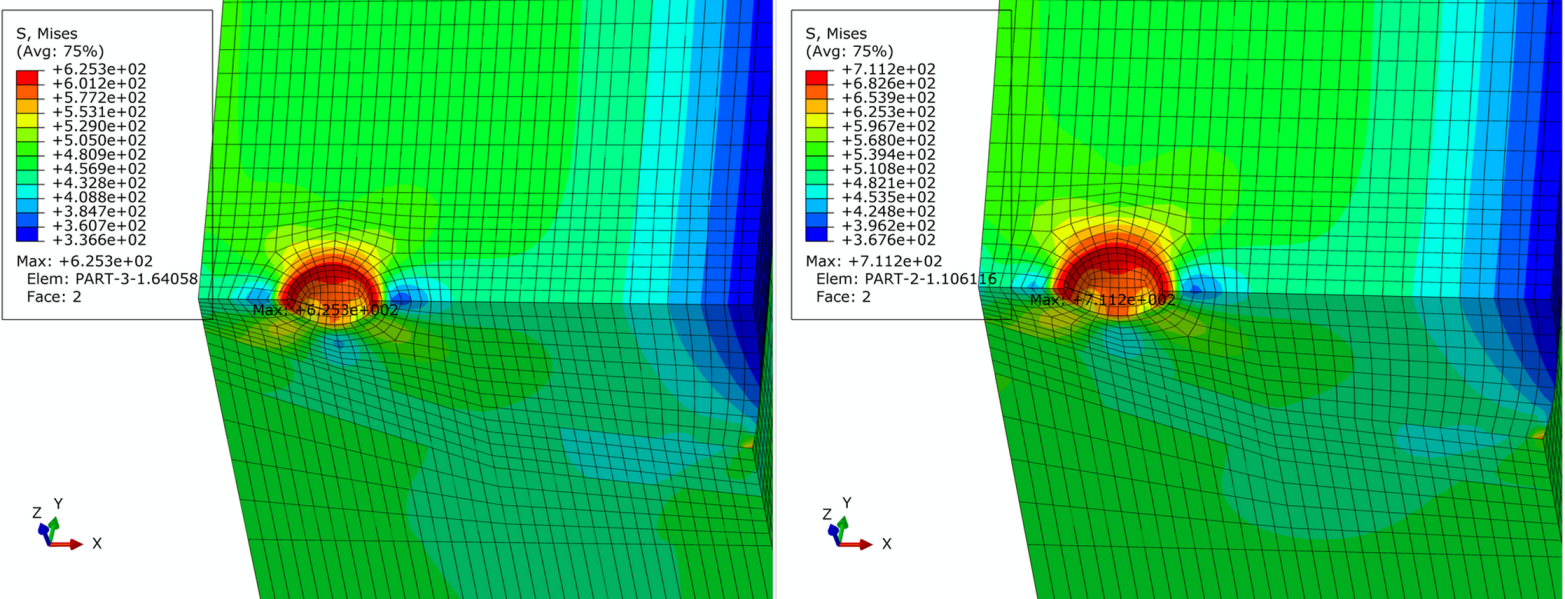
von Mises stress distribution of pipe with inner pore under extremely tensile loading condition. (A) API 5L X80 grade pipe. (B) API 5L X90 grade pipe.

**Fig 12:**
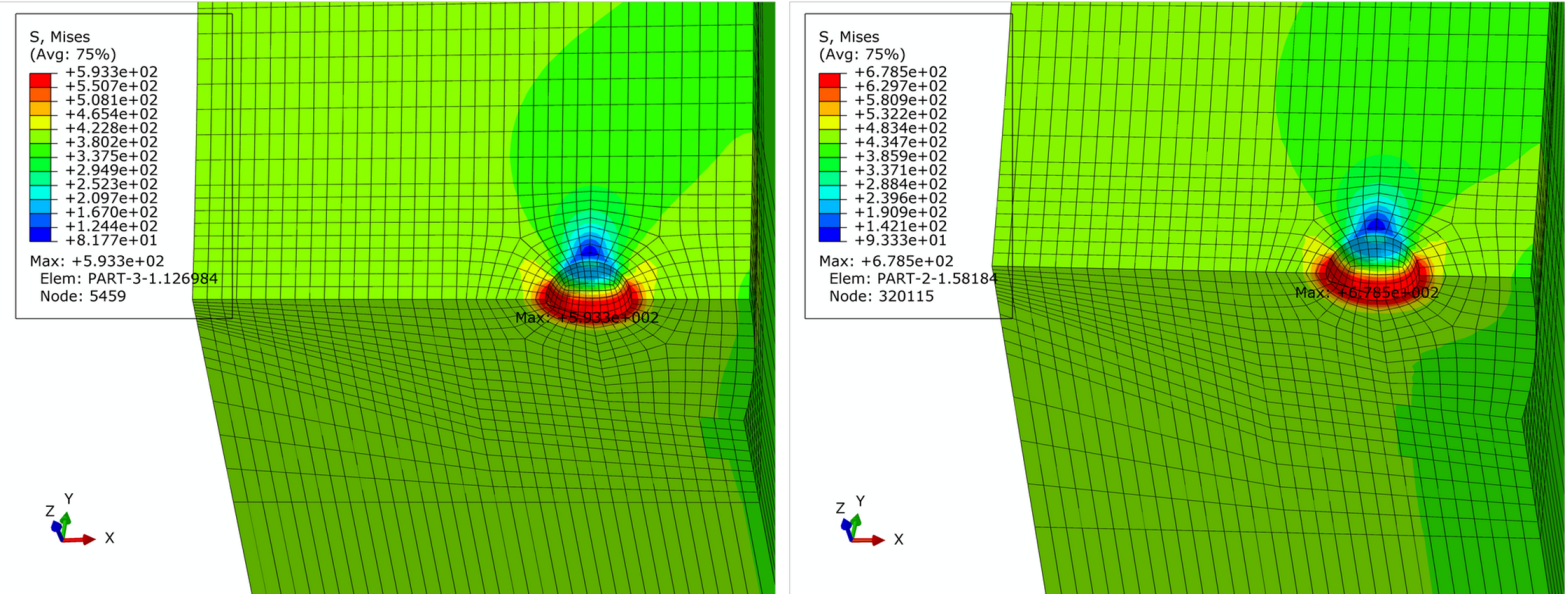
von Mises stress distribution of pipe with outer pore under basic loading condition. (A) API 5L X80 grade pipe. (B) API 5L X90 grade pipe.

**Fig 13:**
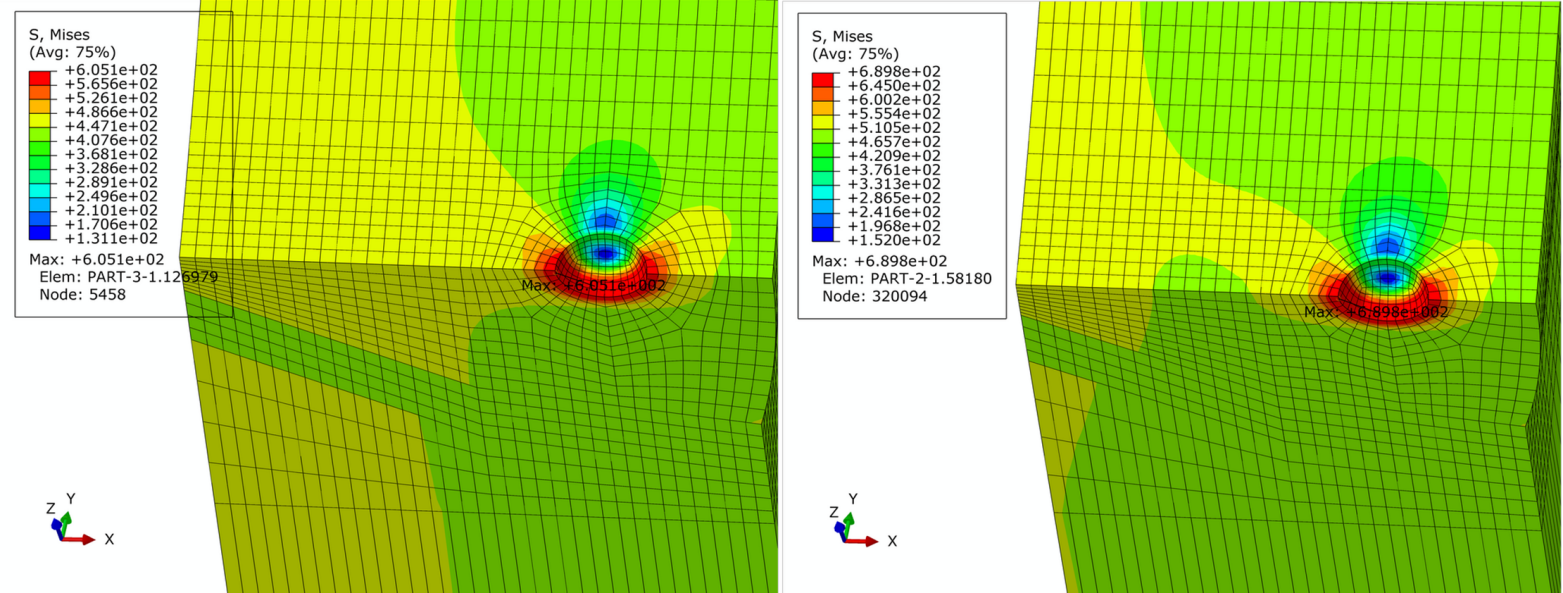
von Mises stress distribution of pipe with outer pore under extremely compressive loading condition. (A) API 5L X80 grade pipe. (B) API 5L X90 grade pipe.

**Fig 14:**
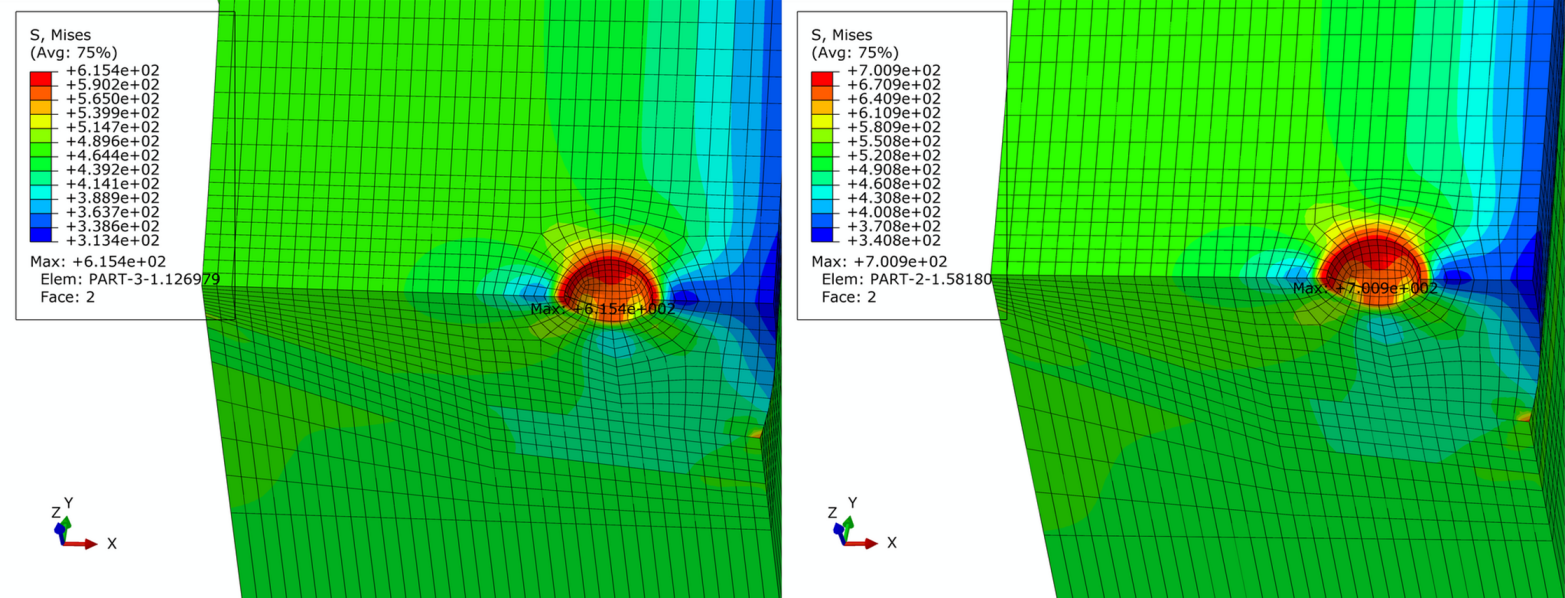
von Mises stress distribution of pipe with outer pore under extremely tensile loading condition. (A) API 5L X80 grade pipe. (B) API 5L X90 grade pipe.

To analyze effect of pore location on pipe stress state, pore defects on both inner and outer sides of the centerline of the pipe cross section are established, with the centerline as the boundary. The finite element models are shown in Fig 8. From Figs 9–14, Pipe stress distributions for both pore defects are similar to that of the weld center pore. Significant stress increase is induced in the region around the pore. However, the affected region is limited without extension to the inner side or outer side of girth weld, and remaining region of pipe is characterized by even distribution of stress without yielding. From results shown in Table 3, it can be observed that maximum equivalent stress of the inner pore is slightly greater than that of the outer pore, but this difference is less than 2%. This demonstrates that pore location change along radial direction has little impact on the distribution of pore local stress and pipe body stress. Based on these results, effect of pore location can be neglected.

**Table 3:** The comparison of von Mises stress.

Loading condition	Pipe grade	von Mises stress/MPa		
		Inter	Middle	Outer
**Basic**	API 5L X80	598.2	595.2	593.3
	API 5L X90	682.9	679.9	678.5
**Extremely compressive**	API 5L X80	607.7	605.8	605.1
	API 5L X90	692.8	690.5	689.8
**Extremely tensile**	API 5L X80	625.3	621.4	615.4
	API 5L X90	711.2	706.4	700.9

### Shape of pore

**Fig 15:**
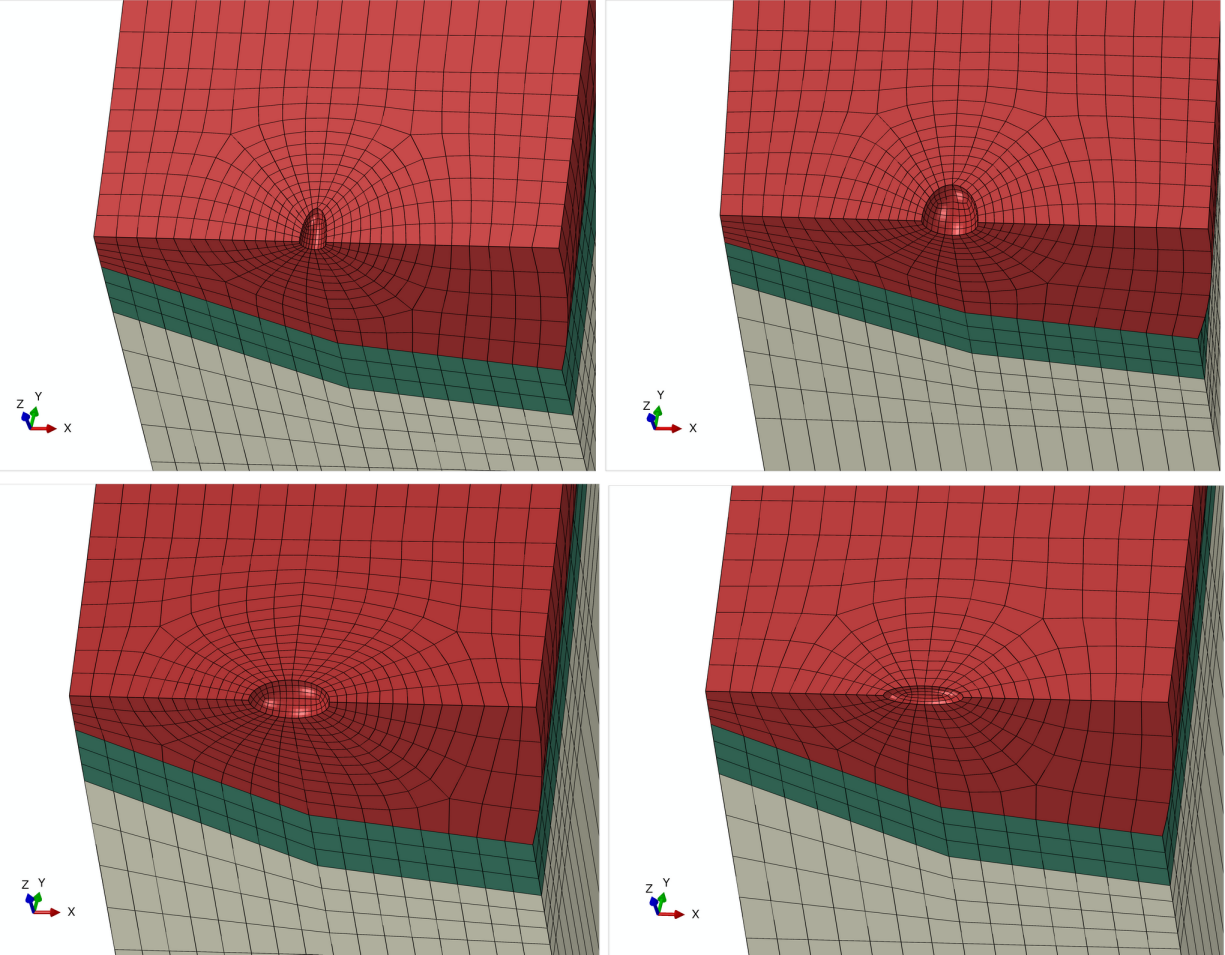
Finite element model of pore defect with different shape. (A) S = 1/3. (B) S = 2/3. (C) S = 3/2. (D) S = 3/1.

Shape of pore can be represented by sharpness. Pore sharpness measures degree of shape sharpness of the pore. Under different loading conditions, pores of different sharpness will induce different local stress distributions in the pipe. To analyze effect of pore shape on pipe stress state, ratio of dimension *H* along the radial direction of the pipe to dimension *W* along circumferential direction is used to denote sharpness of pore *S*, i.e. *S* = *H*/*W*. Therefore, in finite element analysis, ellipsoidal defect can be used to approximate pores of different sharpness. Since, pore location has no impact, ellipsoidal defect is established at the center of weld. Fig 15 show the finite element models of pipes with pore defects with four different sharpness.

**Fig 16:**
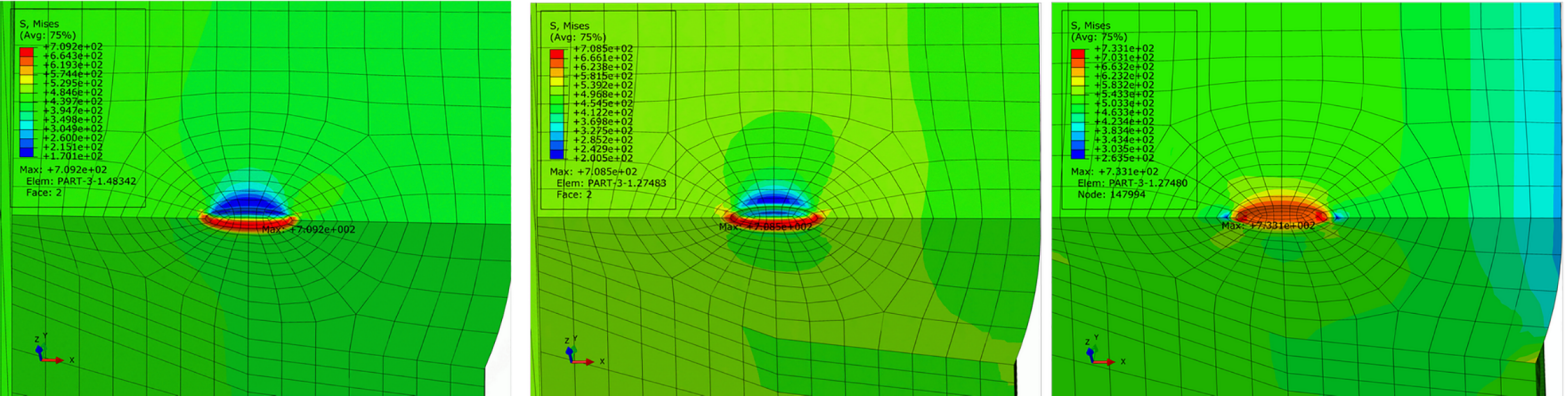
von Mises stress distribution of pipe with sharp pore (S = 3/1). (A) Basic loading condition. (B) Extremely compressive loading condition. (C) Extremely tensile loading condition.

Stress variation in pipes with ellipsoidal pore defect is similar to that with sphere defect. However, different pore sharpness results in different stress concentration degrees around the pore. Affected area is limited without extension to either the inner side or outer side of pipe, and so overall stress state of pipe remains uniform. Simulation results of X90 pipe are shown in Table 4. Comparison results reveal that increase in pore sharpness results in increase in maximum equivalent stress at the defect. When, major axis of the ellipsoidal sharp defect is along the radial direction of pipe (S = 3/1), maximum equivalent stress at the defect is significantly greater than that in pipe models with pore with other sharpness values. This is especially the case under combined loading of internal pressure and axial tensile stress, as shown in Fig 16, obvious stress concentration is observed around the limited region of pore defect and the maximum equivalent stress of pipe exceeds flow stress but has not reached strength limit. Although the stress level of most part is below yield stress, the locally high stress concentration area may lead to crack propagate under large pressure fluctuations. From this perspective, this kind of defect is not acceptable and should be paid particular attention for safe operations.

**Table 4:** Comparison of pore defect with different sharpness.

von Mises stress/MPa Loading condition	Pipe grade	S = H/W				
		1/3	2/3	3/3	3/2	3/1
**Basic**	API 5L X80	482.1	578.2	595.2	647	708.3
	API 5L X90	550.5	639.3	674.9	684.3	709.2
**Extremely compressive**	API 5L X80	566.5	593.4	605.8	647.8	684.4
	API 5L X90	637.6	665.5	686.1	694.3	708.5
**Extremely tensile**	API 5L X80	729.2	652	621.4	708.6	821.5
	API 5L X90	706	695.1	709.5	709.7	733.1

### Material grade

Table 5 lists the comparison of maximum equivalent stresses of API 5L X80 and X90 grade pipes with pore defect in girth weld and flow stress. As pipe grade increases, outer diameter of pipe increases and wall thickness decreases, causing increase in hoop stress of the pipe. A combined effect of these factors suggests that tolerance of X90 pipe girth weld to pore defect is lower than that of API 5L X80 grade pipe girth weld and stress is close to the reference value of flow stress determined by failure criteria.

**Table 5:** Comparison of stress ratio.

Loading condition	Pipe grade	von Mises stress/flow stress						
		Inner	Middle	Outer	S = 1/3	S = 2/3	S = 3/2	S = 3/1
**Basic**	API 5L X80	0.925	0.921	0.919	0.743	0.891	0.937	0.961
	API 5L X90	0.944	0.940	0.925	0.758	0.881	0.943	0.977
**Extremely compressive**	API 5L X80	0.938	0.935	0.934	0.873	0.910	0.947	0.961
	API 5L X90	0.956	0.953	0.952	0.878	0.917	0.956	0.976
**Extremely tensile**	API 5L X80	0.968	0.964	0.958	0.964	0.945	0.952	0.986
	API 5L X90	0.983	0.978	0.973	0.973	0.957	0.978	1.009

## Conclusions

In the present work, common pore defect in pipe girth weld, based on real weld groove size used as the guideline for modeling, combined with softening material in heat-affected zone, a three-dimensional finite element model is established to study effect of pore defect on stress distribution of pipe girth weld under three loading conditions. The following conclusions were reached based on the results.

1. Pore defect leads to increased local stress in weld, and location is associated with loading condition, but it is not extended to inner or outer sides of the girth weld. Under basic or extremely compressive loading conditions, hoop stress induced by internal pressure prevails, and increase in stress around the edge of the pore on vertical plane is most significant. Under extremely tensile loading condition, when effect of tensile stress is dominant compared to hoop stress induced by internal pressure, most significant stress location moves to the pore edge on the cross section.
2. Stress analysis revealed that for pores located on the inner side, outer side and center of pipe wall, change in radial location of pore does not significantly affect stress level around the pore, so impact of radial location of pore is negligible.
3. Stress analysis for pore defects of various sharpness revealed that influence of pore shape on stress state of pipe is relatively significant; especially for pore defect of relatively large sharpness, stress at defect significantly grows under tensile loading condition and exceeds flow stress, to which attention should be paid.
4. As pipe grade increased, outer diameter increased and wall thickness decreased, and hoop stress level of pipe is increased owing to these changes in material and geometry dimensions. Combined effect of these factors results in reduced acceptable level of defect in high-grade pipes.
5. Only dimension of the major axis of pore defect is prescribed in current standards, and the impact of shape of defect is not considered. It was found that API 5L X80 and X90 grade pipes with overmatched girth weld and a design parameter of 0.72 under extreme loading conditions, stress of pipes with sharpness of pore defect *S* ≤ 3/2 are below flow stress prescribed by the failure criteria. Therefore, size control index of a pore defect of 3 mm is applicable only to API 5L X80 and X90 grade pipes with defect that is not radially sharp.

## Supporting information

S1 File The modeling file of finite element model. (CAE)

S2 File The detailed description of finite element model. (DOCX)
