# Supplementary material for: Investigation on size tolerance of pore defect of girth weld pipe
Source: PLoS One. 2018 Jan 24;13(1):e0191575. doi: 10.1371/journal.pone.0191575 (PMC5783411; doi:10.1371/journal.pone.0191575)
Supplement: S2 File — (DOCX) [file pone.0191575.s002.docx]

The numerical simulations were performed by ABAQUS Finite Element Analysis software. The models are modeled as a quarter of girth weld pipe with a pore defect on the symmetry axis. Here we list all the data and setting methods of Finite Element models as following. The modeling file entitled “S1 file.cae” have been submitted as a Supporting Information file.

1. The dimension of finite element models are list in Table 1. Choose “Deformation Plasticity” as the material constitutive model and corresponding parameters are list in Table 2.

**Table1. Pipe dimensions of finite element model**

| **Pipe grade** | **Pipe diameter/mm** | **Wall thickness/mm** |
| --- | --- | --- |
| **API 5L X80** | 1016 | 18.4 |
| **API 5L X90** | 1219 | 16.3 |

**Table2. Parameters of the Ramberg-Osgood model of finite element model**

| **Pipe grade** | **Material type** | **Young’s Modulus/MPa** | **Poisson’s Ratio** | **Yield Stress/MPa** | **Exponent** |
| --- | --- | --- | --- | --- | --- |
| **API 5L X80** | Heat affected zone | 200000 | 0.3 | 499.5 | 23.74 |
|  | Base material | 200000 | 0.3 | 555 | 24.47 |
|  | Weld | 200000 | 0.3 | 610.5 | 25.31 |
| **API 5L X90** | Heat affected zone | 200000 | 0.3 | 562.5 | 26.72 |
|  | Base material | 200000 | 0.3 | 625 | 27.79 |
|  | Weld | 200000 | 0.3 | 687.5 | 29.08 |

1. Step setting: Using the “static, General” algorithm and open the Nlgeom calculation option.
2. Loading conditions:

For loads in each model were applied as monotonically increasing way, where internal pressure remains normal to the pipe internal surface (Fig 1a) and axial pressure remains normal to the pipe end ( Fig 1b).

1. Boundary conditions:

Boundary conditions were applied in cylindrical coordinate system. Two symmetric displacement constraints were applied to the quarter models, in the cross (Fig 2a) and axial section (Fig 2b) planes. In order to eliminate the effect of rigid displacement of models, hoop displacement constraint was applied on pipe end (Fig 1b).


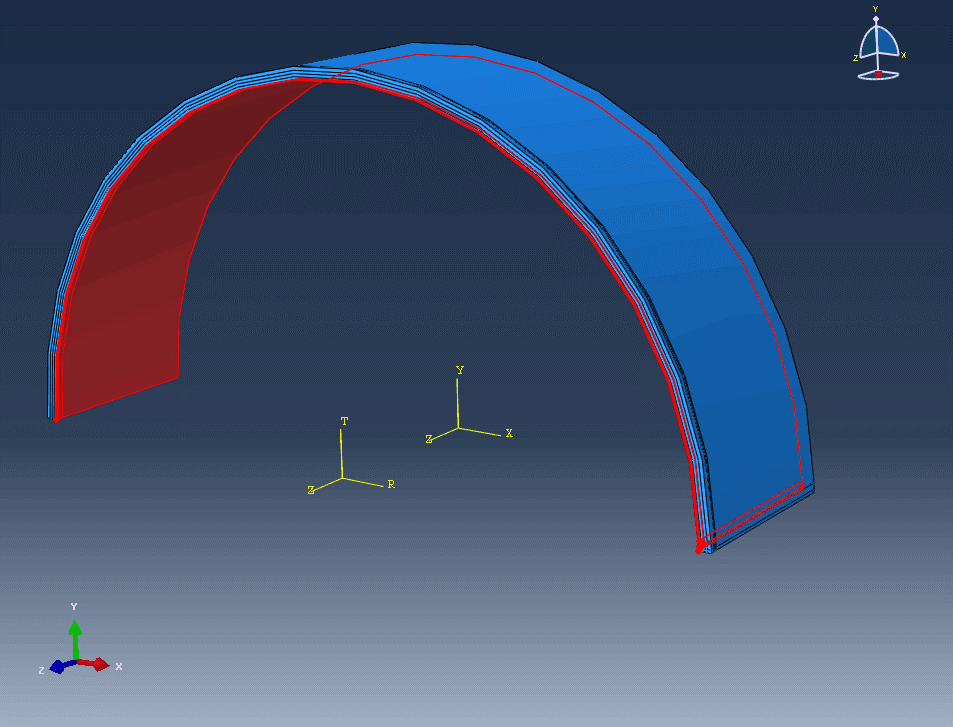

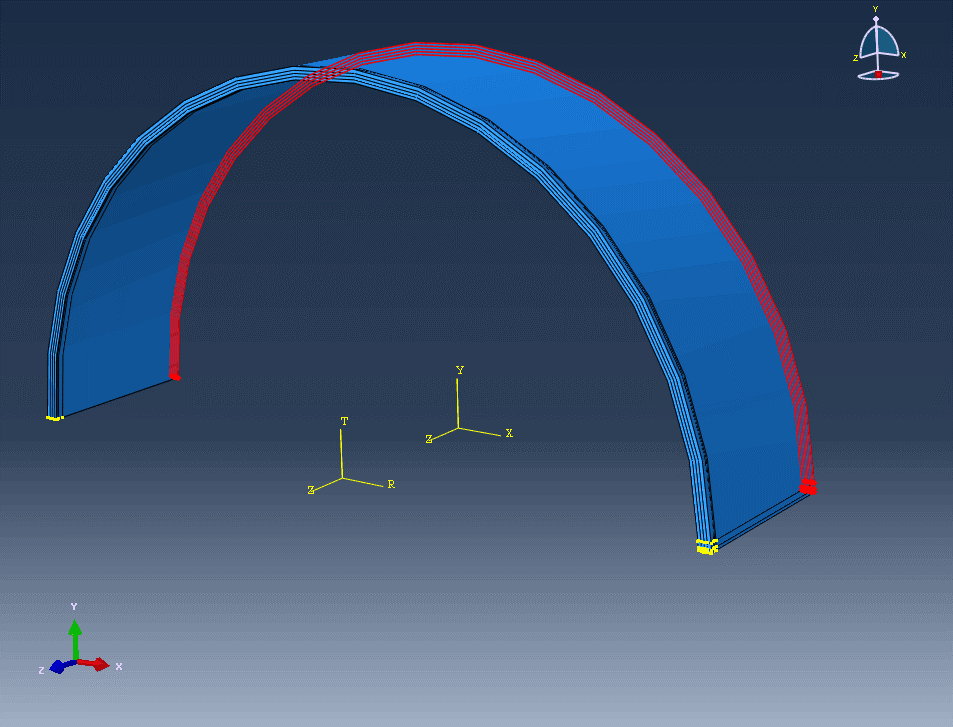


**Fig 1. loading conditions.** (a) internal surface (b) pipe end


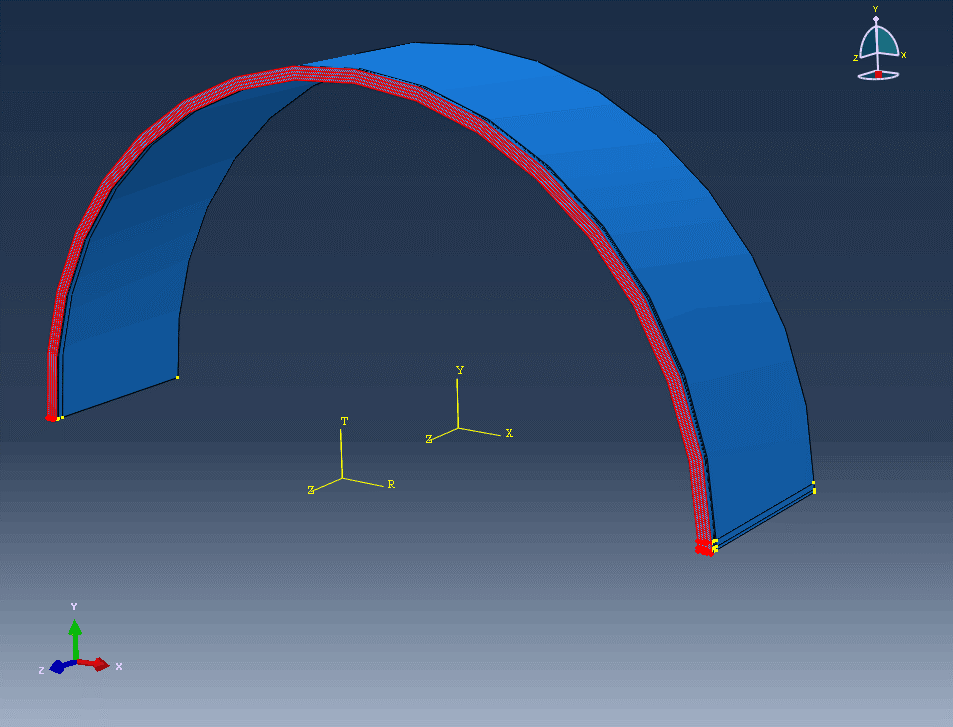

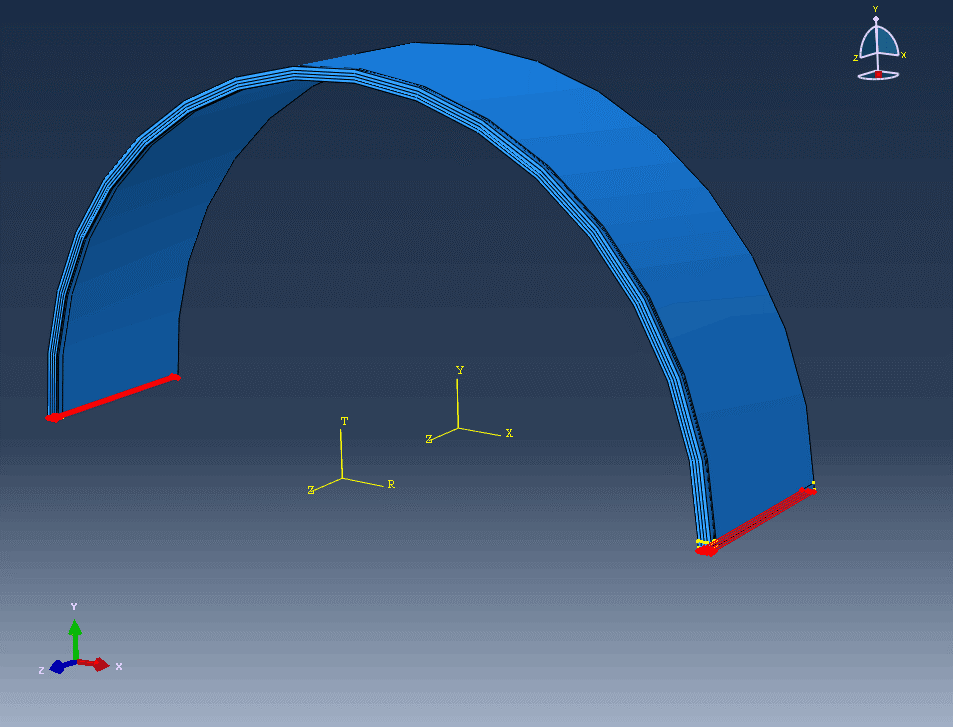


**Fig 2. boundary condition constraints of finite element model.** (a) cross section (b) axial section

1. Meshing：

The element type is C3D20R and the detailed meshing number of each edge was shown in Fig 3.


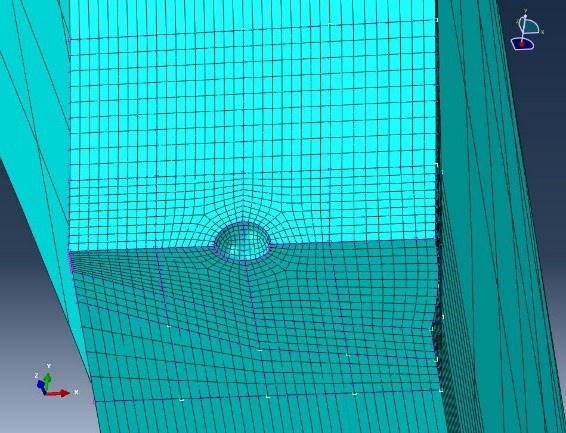

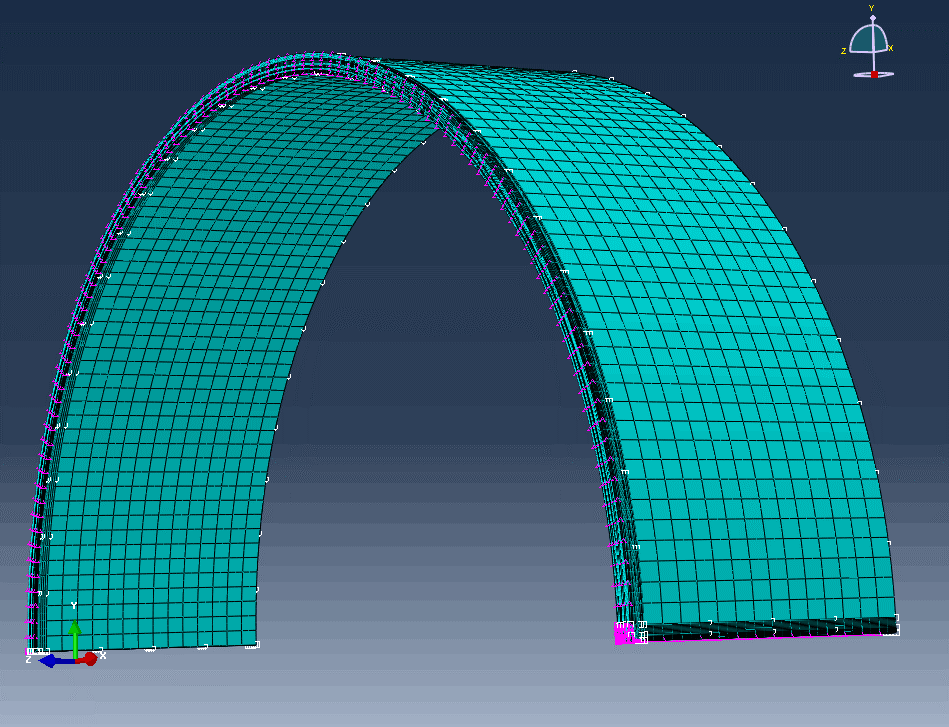


**Fig 3. mesh generation of finite element model.** (a) refined mesh (b) whole model
